# The emerging role of heterodimerisation and interacting proteins in ghrelin receptor function

**DOI:** 10.1530/JOE-21-0206

**Published:** 2021-10-18

**Authors:** Maria L Price, Cameron D Ley, Caroline M Gorvin

**Affiliations:** 1 Institute of Metabolism and Systems Research and Centre for Endocrinology, Diabetes and Metabolism, University of Birmingham, Birmingham, UK; 2 Centre of Membrane Proteins and Receptors (COMPARE), Universities of Birmingham and Nottingham, Birmingham, UK

**Keywords:** appetite, receptor cross-talk, dopamine receptors, GPCR, heterodimerisation, MRAP2

## Abstract

Ghrelin is a peptide hormone secreted primarily by the stomach that acts upon the growth hormone secretagogue receptor (GHSR1), a G protein-coupled receptor whose functions include growth hormone secretion, appetite regulation, energy expenditure, regulation of adiposity, and insulin release. Following the discovery that GHSR1a stimulates food intake, receptor antagonists were developed as potential therapies to regulate appetite. However, despite reductions in signalling, the desired effects on appetite were absent. Studies in the past 15 years have demonstrated GHSR1a can interact with other transmembrane proteins, either by direct binding (i.e. heteromerisation) or via signalling cross-talk. These interactions have various effects on GHSR1a signalling including preferential coupling to one pathway (i.e. biased signalling), coupling to a unique G protein (G protein switching), suppression of GHSR1a signalling, and enhancement of signalling by both receptors. While many of these interactions have been shown in cells overexpressing the proteins of interest and remain to be verified in tissues, substantial evidence exists showing that GHSR1a and the dopamine receptor D1 (DRD1) form heteromers, which promote synaptic plasticity and formation of hippocampal memory. Additionally, a reduction in GHSR1a-DRD1 complexes in favour of establishment of GHSR1a-Aβ complexes correlates with Alzheimer’s disease, indicating that GHSR1a heteromers may have pathological functions. Herein, we summarise the evidence published to date describing interactions between GHSR1a and transmembrane proteins, discuss the experimental strengths and limitations of these studies, describe the physiological evidence for each interaction, and address their potential as novel drug targets for appetite regulation, Alzheimer’s disease, insulin secretion, and inflammation.

## Introduction

Ghrelin is a 28-amino acid gut-derived hormone, referred to as the ‘hunger hormone’, due to its role in stimulating appetite and initiating food intake (Kojima *et al.* 1999). Ghrelin is secreted in an inactive form primarily by the stomach. Activation requires acylation by the ghrelin-O-acyltransferase (GOAT) enzyme, which is itself released under fasted conditions (Gutierrez *et al.* 2008). Ghrelin acts by binding to the growth hormone secretagogue receptor-1 (GHSR1), a G protein-coupled receptor (GPCR) expressed in the brain (primarily on hypothalamic and hippocampal neurons) and endocrine tissues (including the pituitary and pancreas) (Howard *et al.* 1996, Kojima *et al.* 1999). Ghrelin receptor functions include effects on growth hormone (GH) secretion, appetite regulation, energy expenditure, adiposity, insulin release, and gastric emptying. GHSR1 exists in two forms; the active, signalling form, GHSR1a, and an inactive form, GHSR1b, a splice variant with transmembrane (TM) helices 6 and 7 deleted (Howard *et al.* 1996). GHSR1 primarily signals via Gα_q/11_-phospholipase C (PLC), to activate intracellular Ca^2+^ (Ca^2+^_i_) mobilisation, and the receptor exhibits considerable constitutive activity. In 2018, an endogenous antagonist and inverse agonist of GHSR1a, liver-expressed antimicrobial peptide 2 (LEAP2), with similar potency to ghrelin, was discovered. LEAP2 prevents ghrelin-mediated effects on food intake, GH release, and maintenance of glucose levels during chronic caloric restriction (Ge *et al.* 2018).

**Figure 1:**
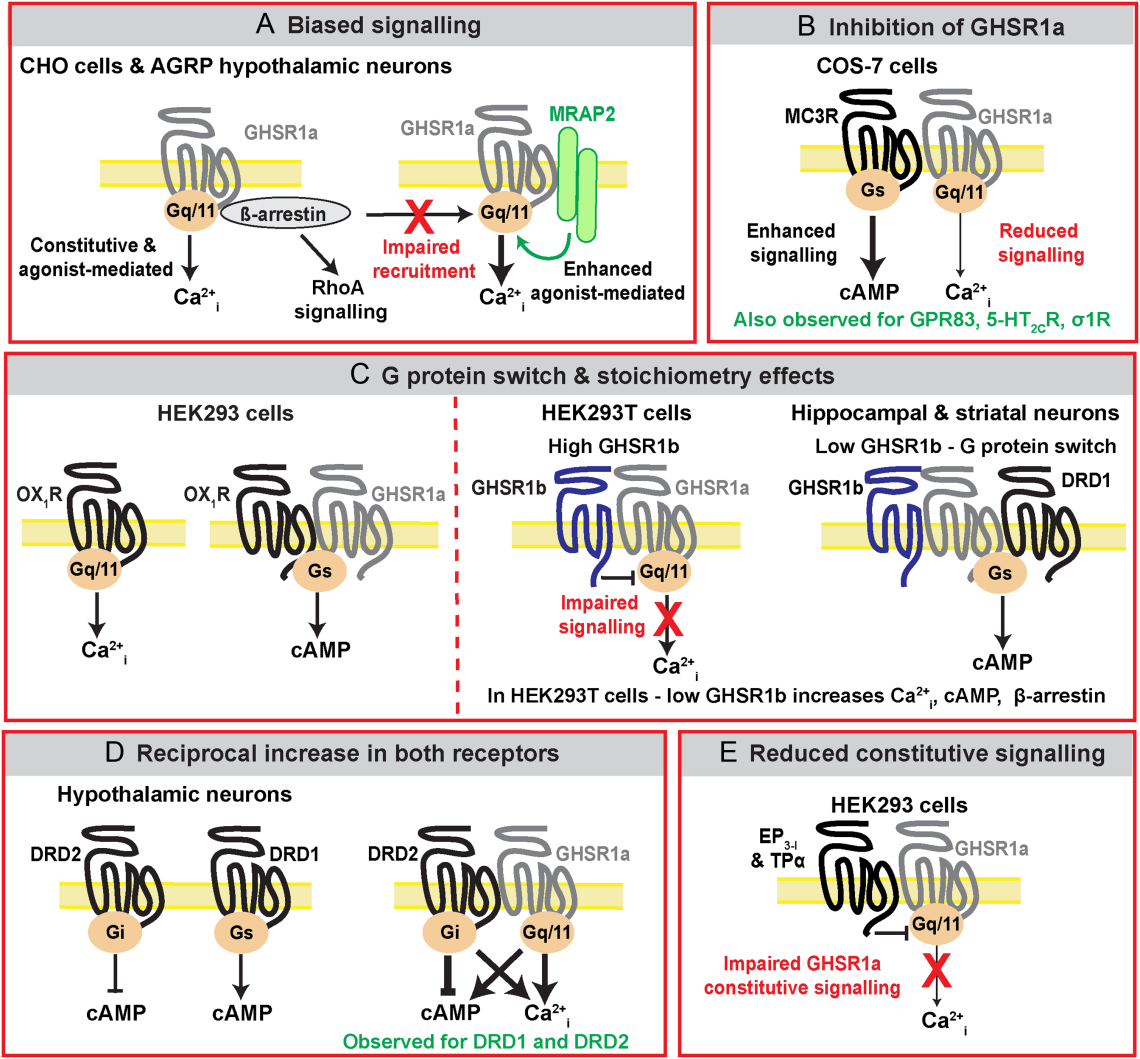
Mechanisms by which GHSR1a signalling is modified by interacting proteins. Schematic showing the different mechanisms by which GHSR1a signalling is modified by interactions with transmembrane proteins. All diagrams depict monomeric receptors, although there is evidence that heterotetramers may occur. GHSR1a is understood to primarily signal via G_q/11_ pathways to activate Ca^2+^_i_ pathways. This involves both constitutive and agonist-mediated signalling, and association between GHSR1a and β-arrestins may increase RhoA pathways. (A) Interaction with MRAP2 impairs GHSR1a constitutive activity and β-arrestin recruitment, and biases signalling towards enhanced agonist-mediated Ca^2+^_i_ signalling. (B) Interactions between GHSR1a and MC3R enhance MC3R-mediated G_s_-cAMP signalling and impair ghrelin-mediated increases in Ca^2+^_i_. Similar mechanisms are observed for GPR83, 5-HT_2C_R, and σ1R. (C) Interactions between GHSR1a and OX1R switch G protein coupling from G_q_ to G_S_. Interactions with GHSR1b and SSTR2 also result in a switch in G protein coupling that is governed by the stoichiometry of each receptor. In the presence of high GHSR1b expression, the inactive receptor exerts a dominant-negative effect on GHSR1a-mediated signalling. When GHSR1b expression is low, as in hippocampal and striatal neurons, GHSR1a interacts with both GHSR1b and DRD1 to activate G_S_-cAMP signalling. (D) Interactions between GHSR1a and DRD2 and DRD1 result in reciprocal increases in signalling by both receptors. (E) Interactions with prostanoid receptors impair GHSR1a constitutive signalling. AGRP, agouti-related peptide; Ca^2+^_i_, intracellular Ca^2+^; DRD1, dopamine receptor D1; DRD2, dopamine receptor D2; EP3-I, prostaglandin E_2_ receptor subtype EP_3-I_; GHSR1, growth hormone secretagogue receptor; GPR83, G protein-coupled receptor-83; MC3R, melanocortin-3 receptor; MRAP2, melanocortin receptor accessory protein-2; OX1R, orexin-1 receptor; TPα, thromboxane prostanoid A2 receptor; 5-HT_2C_R, serotonin (5-hydroxytryptamine) subtype-2C receptor; σ1R; neuronal sigma-1 receptor.

Following the discovery that GHSR1a stimulates food intake, research focused on developing antagonists to the receptor as therapies to regulate appetite. However, despite reductions in signalling when used in cell-based assays, these GHSR1a antagonists reduced GH secretion, but stimulated appetite in rats and dogs (Costantini *et al.* 2011, Hassouna *et al.* 2013). This is perhaps not unexpected given the complex role of GHSR1a in adiposity and energy expenditure that is now understood (Muller *et al.* 2015), and it is likely that different strategies will be required to target GHSR1a. In the past 15 years, new insights into the complexity of GHSR1a signalling have emerged that could yield novel therapeutic targets. These include the identification of interacting proteins, such as the melanocortin receptor accessory protein-2 (MRAP2), and increasing evidence that GHSR1a can form heteromers with several GPCRs, resulting in modification of GHSR1a signalling (Fig. 1 and Tables 1, 2). The ability of GHSR1a to modify signalling when dimerised with other receptors, even in the absence of ligand, could help explain why GHSR1a is widely expressed, including in some brain regions that are inaccessible to circulating ghrelin (Perello *et al.* 2019). In such cells, GHSR1a may act to regulate the functioning of other GPCRs, rather than to mediate ghrelin-specific actions. The potential to target GHSR1a heteromeric complexes could provide new avenues to reduce GHSR1a signalling in specific target tissues to modify ghrelin-dependent and -independent functions. This review will describe the GHSR1a interactions identified to date, and explore their feasibility as therapeutic targets.

**Table 1:** Reported protein interactions with GHSR1a.

Interacting proteins	Cell lines/models	Techniques used to confirm interactions	Effect on GHSR1a signalling	References
MRAP2	Overexpression in CHO. MRAP2-global and AGRP conditional knockout mice.	Co-immunoprecipitation, NanoBiT	Potentiates ghrelin-mediated G_q_ signalling by impairing constitutive activity. Reduces β-arrestin recruitment and impairs β-arrestin-mediated RhoA signalling. Regulates GHSR1a activity in pancreatic δ-cells to facilitate ghrelin-mediated inhibition of insulin secretion.	(Srisai *et al.* 2017, Rouault *et al.* 2020, Yin *et al.* 2020)
SSTR5	INS-1 and HEK293 overexpressing SSTR5 and GHSR1a	TR-FRET in INS-1 cells	GHSR1a-SSTR5 heteromers impair somatostatin responsiveness in favour of ghrelin responses. However, more recent studies have shown that GHSR1a is not expressed in pancreatic β-cells and it is unlikely the two receptors interact in the pancreas.	(Tong *et al.* 2010, Park *et al.* 2012, Adriaenssens *et al.* 2016)
MC3R	Overexpression in HEK293 and COS-7.	Co-localisation by confocal microscopy, ELISA, FRET	Enhances MC3R-mediated cAMP signalling and inhibits GHSR1a ligand-induced and basal signalling. MC3R signalling is favoured when GHSR1a is in its constitutively active state.	(Rediger *et al.* 2009, 2011, Schellekens *et al.* 2013)
GPR83	Overexpression in HEK293 and a mouse hypothalamic N41 cell-line.	ELISA, YFP-protein complementation assay	Reduces GHSR1a-mediated IP3 signalling.	(Muller *et al.* 2013)
5-HT_2C_R	HEK293 stably expressing GHSR1a. Hypothalamic and hippocampal rat neurons.	Co-localisation by confocal microscopy	Reduces ghrelin-mediated calcium influx.	(Schellekens *et al.* 2013, 2015)
OX_1_R	Overexpression in HEK293.	Co-localisation by confocal microscopy, BRET, FRET, co-immunoprecipitation, bimolecular fluorescence complementation	Ghrelin enhances cAMP activity when receptors are co-expressed by increasing G_s_ signalling. G_q_ and G_i_ signalling is unaffected.	(Xue *et al.* 2018)
DRD1	HEK293 overexpressing the receptors, SK-N-SH neuronal cell-line. Hippocampal brain slices, primary hippocampal neurons. Studies in *Ghsr1-/-* mice.	Co-localisation by fluorescence deconvolution microscopy, co-immunoprecipitation, BRET, single-molecule TIRF, PLA	Ghrelin and dopamine enhance cAMP signalling. Dopamine enhances Ca^2+^_i_ mobilisation when in dimers by coupling to Gq-PLC. GHSR1a-DRD1 signalling increases: - phosphorylation of CaMKII, AMPAR, and activates synaptic plasticity;- exocytosis of glutamate receptors in hippocampal neurons- gene transcription to initiate neuronal plasticity.	(Jiang *et al.* 2006, Kern *et al.* 2015, Damian *et al.* 2018)
DRD2	Overexpression of GHSR1a in SH-SY5Y neuroblastoma and HEK293 cells. Primary hypothalamic neurons. SMALPs.	TR-FRET in HEK293 and hypothalamic tissue. FRET in SMALPs.	Dopamine agonists induce Ca^2+^_i_ signalling, which is suppressed by antagonists of either receptor. In SMALPs both receptors engage with their respective G proteins. GHSR1a reduces DRD2-mediated inhibition of Ca_v_2.2 currents.	(Kern *et al.* 2012, Damian *et al.* 2018) (Cordisco Gonzalez *et al.* 2020)
GHS-R1b	HEK293 & HEK293T overexpression. Lipid nanodiscs	BRET, immunoprecipitation, sucrose-density gradients	Reduce intracellular calcium, pERK and β-arrestin signalling. D1R-GHSR1a-GHSR1b form complexes which induce a switch from G_i_ to G_s_ coupling.	(Leung *et al.* 2007, Mary *et al.* 2013, Navarro *et al.* 2016)
σ1R	HEK293T Primary cultures of striatal neurons	Immunocytochemistry, BRET in HEK293T PLA in primary cultures of striatal neurons	Cocaine suppresses GHSR1a-mediated reductions in cAMP and phosphorylation of ERK1/2.	(Aguinaga *et al.* 2019)
OTR	HEK293	Co-localisation by confocal microscopy, FRET	Reduces oxytocin-induced Ca^2+^_i_ signalling. Confocal microscopy indicates that the two receptors may co-internalise.	(Wallace Fitzsimons *et al.* 2019)
Prostanoid receptors (EP_3-I_ and TPα receptor)	HEK293	BRET, co-immunoprecipitation	Reduces total GHSR1a expression, increases GHSR1a intracellular localisation and reduces GHSR1a constitutive activity	(Chow *et al.* 2008)

AMPAR, α-amino-3-hydroxy-5-methyl-4-isoxazolepropionic acid receptor; BRET, bioluminescence resonance energy transfer; Ca^2+^_I_, intracellular calcium; CaMKII, calcium/calmodulin-dependent protein kinase II; Ca_v2.2_, N-type voltage-gated calcium channel; ERK1/2, extracellular signal-regulated kinase; FRET, Förster resonance energy transfer; IP3, inositol trisphosphate; PLA, proximity ligation assay; PLC, phospholipase C; SMALP, styrene maleic acid copolymer membrane discs; TIRF, total internal reflection fluorescence; TR-FRET, time resolved-FRET; YFP, yellow fluorescent protein.

**Table 2:** Summary of studies in which GHSR1a interactions with transmembrane proteins have been investigated in physiological systems.

Interacting proteins	Cell lines/models	References
MRAP2	In MRAP2-knockout mice: - ghrelin-induced AMPK phosphorylation and cFos activity are reduced - ghrelin-mediated increases in food intake are absent In AGRP-neuron conditional MRAP2 knockout mice: - GCamp6 calcium oscillations are impaired in AGRP neurons In conditional mice with deletion of MRAP2 from somatostatin-secreting cells: - ghrelin-mediated inhibition of insulin secretion is abolished.	(Srisai *et al.* 2017, Yin *et al.* 2020)
GPR83	- Acute food intake is significantly higher in *Gpr83*^−/−^ mice following a single ghrelin injection. - Twice-daily ghrelin treatment of high-fat-diet fed mice for 6 days showed a greater increase in food intake in *Gpr83*^−/−^ mice. - *Gpr83−/−* mice had increased body weight, food intake, and fat mass following ghrelin infusion when fed regular chow.	(Muller *et al.* 2013)
5-HT_2C_R	- Administration of a 5-HT_2C_R antagonist enhanced ghrelin-induced increases in food intake, and extended the duration of ghrelin’s orexigenic effect. - Administration of lorcaserin delayed ghrelin-induced effects on food intake.	(Schellekens *et al.* 2015)
DRD1	*Ghsr−/−* hippocampal slices showed GHSR1a-DRD1 signalling is required for: - phosphorylation of CaMKII, AMPAR - activation of synaptic plasticity; - exocytosis of glutamate receptors in hippocampal neurons; - gene transcription for initiation of neuronal plasticity. Behavioural studies showed: - DRD1-selective agonists disrupted prepulse inhibition. Prepulse inhibition is disrupted in *Ghsr+/+* but not *Ghsr−/−* mice when pre-injected with a DRD1 agonist. - Mice treated with DRD1-specific agonist SKF81297 had a significant decrease in freezing behaviour in contextual and cued-fear conditioning apparatus, which was impaired by co-administration with a GHSR1a antagonist. - Mice infused with SKF81297 continued to make correct choices in a T-maze alternation test, which was blocked by co-administration with a GHSR1a antagonist, consistent with enhanced memory when GHSR1a-DRD1 heteromers are activated. In GHSR1a-null, 5xFAD and double mutant GHSR1a-null/5xFAD mice there were: - similar reductions in synapse density; - impaired stimulus-evoked LTP in hippocampal slices; - impaired spatial navigation in the Morris water maze test, indicating that the same pathways are affected in mice. Restoration of GHSR1a-DRD1 complexes, by simultaneous stimulation of GHSR1a and DRD1 with MK0677 and SKF81297: - increased synaptic density in Aβ42-treated mouse hippocampal neurons; - improved stimulus-evoked LTP, and restored miniature excitatory postsynaptic current frequency in 5xFAD mice; - reversed memory defects in the Morris water maze test and reduced hippocampal synapse loss in 5xFAD mice.	(Kern *et al.* 2015)
DRD2	- Agonists of DRD2 and GHSR1a stimulated calcium in primary hypothalamic neurons. - The DRD2 selective agonist cabergoline suppressed food intake in WT mice. - Pre-treatment with the GHSR1a antagonist JMV2959 prevented the cabergoline effect on food intake.	(Kern *et al.* 2012)
GHS-R1b	In striatal neurons – a DRD1 antagonist blocked ghrelin-induced Gs-mediated cAMP accumulation.	(Navarro *et al.* 2016)
σ1R	In primary striatal neurons – impaired GHSR1a-mediated signalling on pre-treatment with cocaine or PRE-084.	(Aguinaga *et al.* 2019)

AGRP, agouti-related peptide; AMPAR, α-amino-3-hydroxy-5-methyl-4-isoxazolepropionic acid receptor; AMPK, AMP-activated protein kinase; CaMKII, calcium/calmodulin-dependent protein kinase II; LTP, long-term potentiation.

## GHSR1a interactions with the melanocortin receptor accessory protein-2 (MRAP2)

MRAP2 is a single transmembrane protein that is highly expressed in the hypothalamus and adrenal gland (Chan *et al.* 2009). It was identified as a regulator of the melanocortin family of GPCRs but has since been shown to interact with other GPCRs involved in energy homeostasis, including the prokineticin receptor-1 and orexin-1 receptor (Chan *et al.* 2009, Sebag *et al.* 2013, Rouault *et al.* 2017). MRAP2 stimulates growth in zebrafish; while deletion of the protein causes severe obesity in mice, and genetic variants are associated with obesity in humans (Asai *et al.* 2013, Sebag *et al.* 2013). Observations that MRAP2-deficient mice ate 30% less food compared to their WT littermates following a 24-h fast, indicated that MRAP2 may also have an important role in hunger sensing and in the starvation-mediated activation of orexigenic agouti-related peptide (AGRP) neurons, similar to GHSR1a (Srisai *et al.* 2017). Indeed, MRAP2-knockout mice had reduced fasting-induced activation of AGRP neurons (decreased cFos activity and failure to induce *Agrp* and neuropeptide Y (*Npy*) gene expression); while mice with specific deletion of MRAP2 from AGRP neurons failed to develop obesity and ate significantly less after a 24-h fast (Srisai *et al.* 2017). This led to the hypothesis that MRAP2 may also interact with GHSR1a, which was confirmed by co-immunoprecipitation and NanoBiT experiments in Chinese Hamster Ovary (CHO) cells (Srisai *et al.* 2017). Furthermore, when MRAP2 is deleted from mice, ghrelin-mediated functions are impaired. Thus, in global knockout mice, ghrelin-induced AMPK phosphorylation and cFos activity were reduced, while ghrelin-mediated increases in food intake were absent. In mice with MRAP2 deleted from AGRP neurons, GCamp6 calcium oscillations were impaired, indicating that MRAP2 potentiates GHSR1a signalling in these neurons.

A subsequent study investigated the mechanism by which MRAP2 enhances GHSR1a signalling in transfected CHO cells. MRAP2 had no effect on GHSR1a surface expression, ghrelin affinity, or guanine nucleotide exchange factor activity (i.e. the ability of the Gα_q_ protein to exchange GDP for GTP) (Srisai *et al.* 2017, Rouault *et al.* 2020). MRAP2 was shown to increase GHSR1a activity by inhibiting the receptor’s constitutive activity, using inositol monophosphate (IP__1__) assays. In cells transfected with increasing concentrations of GHSR1a, IP_1_ responses increased in the absence of ligand, but when MRAP2 was co-expressed, this constitutive activity was lost (Rouault *et al.* 2020). MRAP2 reduced β-arrestin recruitment in NanoBiT assays and impaired β-arrestin-mediated RhoA signalling (Rouault *et al.* 2020). Thus, MRAP2 elicits signalling bias upon the GHSR1a, by enhancing G_q__/11_ signalling and reducing β-arrestin pathways (Fig. 1).

These studies indicate that compounds that disrupt the GHSR1a and MRAP2 interaction sites, may be more effective in reducing food intake than those targeting GHSR1a alone. Modifying MRAP2 abundance would be unlikely to be effective, as MRAP2 regulates other GPCRs. The finding that GHSR1a constitutive activity is lost upon interaction with MRAP2 implies that GHSR1a agonist-independent signalling may not be physiologically relevant, at least in cells expressing MRAP2. A number of previous studies have provided evidence that GHSR1a constitutive activity has a physiological function; however, interpretation of some of these data can be problematic as some GHSR1a-targeting compounds and receptor mutations that impair constitutive activity also affect agonist-driven activity. GHSR inverse agonists inhibit constitutive activity, reduce food intake and body weight in rats, and decrease compensatory hyperphagia following a fast in mice (Petersen *et al.* 2009, Fernandez *et al.* 2018). However, these compounds may also elicit antagonistic properties and therefore the interpretation of their effects is problematic. Moreover, a number of studies have investigated the GHSR1a-A204E variant, which has a loss of constitutive activity *in vitro*, and has been identified in patients with short stature and obesity (Pantel *et al.* 2006). Although this mutation abolishes constitutive activity, reduces body length, and impairs GH release in mice with the equivalent A203E mutation, agonist-mediated activity was also impaired in these models, implying that the phenotypes observed in human carriers of the GHSR1a-A204E mutation cannot be attributed solely to defective constitutive activity (Torz *et al.* 2020). Similarly, studies of the GHSR1a-A204E mutation with MRAP2 showed loss of constitutive activity but also reduced ghrelin-mediated responses, and structural studies suggest A204 may regulate ghrelin’s access to binding cavities (Rouault *et al.* 2020, Shiimura *et al.* 2020). Thus, studies with A204E should be interpreted cautiously, as the mutation also impairs ghrelin-mediated activity. Further studies are likely required to determine the physiological role of GHSR1a basal activity in the regulation of energy metabolism.

MRAP2 has also been shown to regulate GHSR1a in pancreatic islets (Yin *et al.* 2020). Ghrelin is known to exert acute inhibition on insulin release and impair glucose tolerance in humans and rodents (Tong *et al.* 2010, Adriaenssens *et al.* 2016). Early studies indicated that GHSR1a may be expressed on both human pancreatic α- and β-cells, and that the receptor suppresses insulin secretion by coupling to G_i__/o_ signalling pathways, rather than canonical G_q_ pathways (Dezaki *et al.* 2007). This G protein switching in pancreatic β-cells was hypothesised to occur via dimerisation between GHSR1a and the somatostatin receptor subtype-5 (SSTR5), and studies in transfected INS-1 cells provided evidence for interaction between the two receptors (using time-resolved FRET (TR-FRET)) and cross-talk between the receptor’s signalling pathways (Park *et al.* 2012). However, more recent RNA-seq studies have shown that GHSR1a is exclusively expressed in pancreatic δ-cells (Adriaenssens *et al.* 2016, DiGruccio *et al.* 2016). These later studies have shown that ghrelin activation of GHSR1a on δ-cells triggers somatostatin release, which subsequently inhibits β-cells by activation of G_i__/o_-coupled SSTRs (Adriaenssens *et al.* 2016, DiGruccio *et al.* 2016). Furthermore, ghrelin infusion of mouse pancreases still stimulated somatostatin secretion in the presence of inhibitors of SSTR2, 3, and 5, but abolished insulin and glucagon release, demonstrating ghrelin actions upon somatostatin are unlikely to occur via direct coupling between the two receptors (Adriaenssens *et al.* 2016). In contrast, deletion of MRAP2 from pancreatic somatostatin-expressing cells of mice prevents the ghrelin-mediated inhibition of insulin secretion, indicating MRAP2 likely directly regulates GHSR1a activity in pancreatic δ-cells (Yin *et al.* 2020). These studies highlight the importance of *in vivo* verification of GHSR1a interactions identified in cell models.

## GHSR1a interaction with melanocortin-3 receptor (MC3R)

MC3R is a GPCR expressed on both pro-opiomelanocortin and AGRP neurons of the hypothalamus, where it has a role in maintaining homeostatic energy set-points, by controlling responses to both calorie restriction and calorie-rich diet (Ghamari-Langroudi *et al.* 2018). Thus, MC3R and GHSR1a share roles in monitoring and maintaining energy balance. When co-transfected in HEK293 cells, GHSR1a and MC3R colocalised, and may internalise together (Rediger *et al.* 2009, Schellekens *et al.* 2013). The two receptors are located within close proximity when overexpressed in COS-7 and HEK293 cells, shown by ELISA and Förster resonance energy transfer (FRET), respectively (Rediger *et al.* 2009). *In situ* hybridisation in mouse hypothalamic brain slices showed that the majority of GHSR-expressing neurons in the arcuate nucleus also express MC3R, but reciprocally, not all MC3R-expressing neurons express GHSR1a (Rediger *et al.* 2011). Heterodimerisation of the two receptors leads to enhancement of MC3R ligand-induced signalling, shown by increased cAMP accumulation, and inhibition of GHSR1a ligand-induced and basal signalling, shown by reduced inositol phosphate (IP) accumulation in COS-7 cells co-transfected with the two receptors (Rediger *et al.* 2011) (Fig. 1). Replication of these studies using a series of receptor mutants showed that dimerisation still occurs, but hyperstimulation of Gs in heterodimers is lost when either a loss-of-function MC3R mutant (Ile183Asn) or mutant GHSR1a with partial loss of constitutive activity (Ala204Glu, Phe279Leu) are introduced (Rediger *et al.* 2011). Moreover, the GHSR1a inverse agonist substance P reduced maximal MC3R cAMP.

These studies indicate that functional MC3R is absolutely required for enhanced cAMP signalling from heterodimers, and that the structure of the GHSR1a, when in the constitutively active state, favours MC3R signalling. Therefore, within these heterodimers GHSR1a must adopt a structural conformation that mediates constitutive activity but has reduced capacity to bind ghrelin, or impaired Gα coupling and/or activation. Investigation of how GHSR1a-MC3R heterodimers interact with G proteins could reveal further insights into how MC3R signalling is enhanced. MC3R heterodimerisation with GHSR1a could be one mechanism by which hypothalamic neurons exert the balance between orexigenic and anorexigenic signals. In a high energy state, heterodimer formation may be favoured, suppressing GHSR1a and increasing MC3R signalling, while low energy states may favour monomers, allowing GHSR1a signalling to proceed. Further investigation in physiologically relevant cells will be required to verify this.

## GHSR1a interaction with G protein-coupled receptor-83 (GPR83)

Interaction between GPR83 and the GHSR1a was shown in 2013, several years before the natural agonist of GPR83 was identified (Muller *et al.* 2013). Studies showed that GPR83 was concentrated within arcuate neurons expressing AGRP and GHSR1a, and that its expression was regulated by nutrient availability, indicating a possible role in appetite regulation (Muller *et al.* 2013). A neuropeptide designated PEN, which is produced from the proSAAS precursor protein, was identified in 2016 as a ligand for GPR83 (Gomes *et al.* 2016). Mice overexpressing proSAAS develop obesity, while proSAAS-null mice exhibit reduced body weight, and antibody-mediated neutralisation of endogenous PEN reduces food intake in mice (Morgan *et al.* 2010, Gomes *et al.* 2016). *Gpr83^−/−^* mice had normal body weight, food intake, and insulin sensitivity when fed a regular diet; but decreased free-fatty acids, body fat mass, and plasma leptin levels compared to controls, indicating GPR83 may be involved in regulating lipolysis (Muller *et al.* 2013). Moreover, *GPR83^−/−^* elicited enhanced reward behaviours (preference for sucrose) and attenuation of stress-evoked anxiety.

Heterodimerisation between GHSR1a and GPR83 was shown in HEK293 cells and a mouse hypothalamic N41 cell-line overexpressing the receptors using ELISA and yellow fluorescent protein-based protein complementation assays. Moreover, co-transfection of cells with these receptors reduced ghrelin-mediated inositol trisphosphate (IP3), indicating that GPR83 may inhibit GHSR1a signalling (Muller *et al.* 2013). Acute food intake was significantly higher in Gpr83^−/−^ mice, when compared to WT littermates, following a single intraperitoneal injection of ghrelin; while twice-daily s.c. ghrelin treatment of high-fat diet-fed mice for 6 days showed a greater increase in food intake in Gpr83^−/−^ mice. *Gpr83^−/−^* mice had increased body weight, food intake, and fat mass following ghrelin infusion when fed regular chow. Therefore, these studies provide evidence that GPR83 may influence GHSR1a-mediated signalling and physiological function. However, *Gpr83^−/−^* mice were protected from diet-induced obesity and glucose intolerance, and plasma levels of acyl-ghrelin and des-acyl-ghrelin were unaffected when fed a high-fat diet for 18 weeks, indicating that GPR83 likely has ghrelin-independent roles in appetite regulation.

These studies suggest an interplay between GPR83 and GHSR1a *in vivo.* However, it is unclear whether heterodimerisation is responsible for these physiological effects as dimers were only shown in transfected cells overexpressing receptors, and thus it is unclear if these heterodimers form in real tissues; further studies may be required to investigate this. Additionally, interpretation of the data is complicated by the overlap between physiological functions, the shared signalling pathways of GPR83 and GHSR1a (both signal by G_q_ and reduced activation could be due to competition for G proteins and downstream mediators), and evidence that both receptors form heteromers with other GPCRs (including both with MC3R). Further studies of GPR83-GHSR1a heterodimers with ligands for both receptors and investigation of signalling by endogenous receptors could reveal further insights into how these receptors regulate food intake.

## GHSR1a interaction with serotonin (5-hydroxytryptamine, 5-HT) subtype-2C receptor (5-HT_2C_R)

The anorexigenic effect of 5-HT on the brain has been known since the 1970s and considerable effort has been made to develop compounds that can act as agonists on 5-HT receptors, with lorcaserin, a selective 5-HT_2C_R agonist, approved by the Food and Drug Administration for use in obesity. Several lines of evidence indicate cross-talk between serotonin- and ghrelin-mediated signalling within neurons. In mice, fasting induces an increase in expression of the 5-HT_2C_R and 5-HT_1B_R genes alongside increases in plasma active ghrelin compared to the fed state; 5-HT receptor agonists (meta-chlorophenylpiperazine and fenfluramine) inhibit the increase in plasma active ghrelin in mice; ghrelin inhibits serotonin release in rat hypothalamic synaptosomes; and administration of the 5-HT_2C_R agonist 5-dimethoxy-4-iodoamphetamine, attenuates ghrelin-induced food intake in rats (Brunetti *et al.* 2002, Nonogaki *et al.* 2006, Currie *et al.* 2010).

Both *in vitro* and *in vivo* studies have investigated heterodimerisation between 5-HT_2C_R and GHSR1a. Initial studies using confocal microscopy showed co-localisation of overexpressed 5-HT_2C_R with GHSR1a in HEK293 cells, and demonstrated that receptors co-internalise, which was enhanced by treatment with ghrelin, but not with MK0677 and 5-HT (Schellekens *et al.* 2013). GHSR1a and 5-HT_2C_R primarily couple to G_q_, complicating studies of heterodimerisation as effects on signalling could be caused by competition between receptors for downstream signal partners. Studies showed that the dose-dependent maximal ghrelin-induced calcium influx was lower in cells which co-express 5-HT_2C_R and GHSR1a, compared to cells that expressed only GHSR1a, and that treatment with the 5-HT_2C_ receptor-specific antagonist (RS102221) fully restored maximal ghrelin and MK0677-induced calcium influx to levels observed in cells transfected with just GHSR1a (Schellekens *et al.* 2013).

In a subsequent study from the same research group, co-localisation between 5-HT_2C_R and fluorescein-ghrelin was shown in a subset of hypothalamic and hippocampal rat neurons, demonstrating heterodimerisation or receptor cross-talk could be possible in these neurons (Schellekens *et al.* 2015). Investigations in mice showed that administration of a 5-HT_2C_R antagonist (SB242084) enhanced ghrelin-induced increases in food intake and extended the duration of ghrelin’s orexigenic effect. Moreover, administration of lorcaserin delayed ghrelin-induced effects on food intake (Schellekens *et al.* 2015). These studies, demonstrating 5-HT_2C_R antagonism results in a long-lasting orexigenic effect, indicate that therapeutic targeting of heterodimers may be useful to increase appetite and food intake, and could potentially be used in the treatment of anorexia nervosa; while conversely, combinations of 5-HT_2C_R agonists with GHSR1a antagonists could reduce appetite, with possible utility for anti-obesity therapies. Further work investigating the effect of 5-HT_2C_R agonism/antagonism on ghrelin-mediated signalling is required in animal models to investigate the long-term effects on food intake and energy metabolism. Furthermore, as the evidence for heterodimerisation is based entirely on overexpression in cell lines, with comparisons made to single transfected cells, further evidence is required to determine whether the physiological effects observed are due to receptor heterodimerisation or signal pathway cross-talk.

## GHSR1a interaction with the orexin-1 receptor (OX_1_R)

Orexin-A and orexin-B are neuropeptides encoded by a single precursor protein, pre-pro-orexin, produced in the lateral hypothalamus, that activate the orexin-1 and orexin-2 Gα_q_-coupled receptors (OX_1_R and OX_2_R, respectively). The orexins act upon receptors expressed in the prefrontal and infralimbic cortex, hippocampus, hypothalamic nuclei, dorsal raphe nucleus, ventral tegmental area, and locus coeruleus where they regulate functions including sleep and wakefulness, feeding and energy expenditure, and reward (Inutsuka *et al.* 2014, Muschamp *et al.* 2014).

A single study described dimerisation between OX_1_R and GHSR1a when overexpressed in HEK293 cells (Xue *et al.* 2018). Co-localisation of the receptors and close proximity were shown using bioluminescence resonance energy transfer (BRET), FRET, co-immunoprecipitation, and bimolecular fluorescence complementation (BiFC) (Xue *et al.* 2018). Ghrelin enhanced cAMP activity (assessed using ELISA, exchange proteins directly activated by cAMP (EPAC) biosensor, and cAMP response element (CRE) luciferase reporter assays) in cells co-expressing GHSR1a and OX_1_R, compared to cells expressing either receptor alone (Xue *et al.* 2018). Orexin-A was unable to induce increased signalling in cells expressing both receptors. BRET assays examining Gα protein interaction with GHSR1a and OX1R dimers showed enhanced BRET ratios when Gα_s_ was stimulated by ghrelin, compared to co-expressed cells stimulated with orexin, and cells expressing either receptor alone. In contrast, Ca^2+^_i_, serum-response element luciferase reporter activity, and BRET ratios with Gα_q_ and Gα_i_ were unchanged in cells co-expressing GHSR1a and OX_1_R (Xue *et al.* 2018). The authors concluded that GHSR1a-OX_1_R heterodimers favour coupling to Gα_s_, rather than Gα_q_ that is utilised by each receptor when expressed alone (Fig. 1).

These studies demonstrated that co-expression of GHSR1a and OX_1_R may allow the receptors to activate novel signalling pathways. It could be hypothesised that this may affect stress responses, food intake, energy homeostasis, and reward behaviours, all of which have been shown to be influenced by ghrelin and orexin. Moreover, MRAP2, which enhances GHSR1a activity in the hypothalamus, negatively regulates OX_1_R (Rouault *et al.* 2017). Therefore, it is possible that MRAP2 could influence signalling by GHSR1a-OX1R dimers. However, heterodimerisation between GHSR1a and OX_1_R was only demonstrated in cells overexpressing the receptors, and therefore the physiological relevance of these findings remain to be demonstrated in neuronal cells and/or animal models.

## GHSR1a interaction with dopamine receptor D1 (DRD1)

GHSR1a has been reported to associate with two dopamine receptors: DRD1 (see GHSR1a interaction with dopamine receptor D2 (DRD2)), which is co-expressed with GHSR1a at the hippocampus, midbrain, substantia nigra, and ventral tegmental areas (Jiang *et al.* 2006); and DRD2, which is co-expressed with GHSR1a at the hypothalamus (Kern *et al.* 2012). DRD1 is a G_s_-coupled receptor which regulates neuronal growth and development, learning and memory, and reward behaviours. Neuromodulation of dopamine signalling by ghrelin was proposed as ghrelin administration to rats dose-dependently augments cocaine-induced hyperactivity, a pathway known to involve dopamine signalling (Wellman *et al.* 2005). Initial studies showed co-treatment of cells overexpressing GHSR1a and DRD1 with dopamine and ghrelin enhanced cAMP signalling by three- to four-fold when compared to cells treated with DRD1 alone (Jiang *et al.* 2006) (Fig. 1). However, dopamine had no effect on ghrelin pathways, measured by aequorin Ca^2+^_i_ signalling. Three methods were used to provide evidence of GHSR1a-DRD1 heteromer formation: co-localisation using fluorescence deconvolution microscopy in a neuronal cell-line (SK-N-SH), immunoprecipitation in lysates from transfected HEK293 cells, and BRET studies which showed enhanced proximity between the receptors when cells were incubated with dopamine and ghrelin (Jiang *et al.* 2006).

The presence of GHSR1a and DRD1 complexes was confirmed in hippocampal brain slices using FITC-labelled ghrelin and a fluorescent DRD1 antagonist (red-SKF83566). The real-time dynamics of DRD1 and GHSR1a interactions were explored in primary hippocampal neuronal cultures using single-molecule total-internal reflection fluorescence microscopy (labelling endogenous proteins). GHSR1a and DRD1 co-localised in membranes, had significantly slower diffusion within complexes, and co-localisation increased in the presence of a DRD1-specific agonist (SKF81297) (Kern *et al.* 2015). In contrast to previous studies (Jiang *et al.* 2006), SKF81297 was shown to dose-dependently induce rapid Ca^2+^_i_ transients in hippocampal brain slices (measured using the Ca^2+^ sensor GCaMP3), which were absent from *Ghsr1^−/−^* mice. Moreover, pre-treatment of hippocampal slices with the GHSR1a antagonist JMV2959 blocked SKF81297-induced Ca^2+^_i_ mobilisation (Kern *et al.* 2015). Dopamine-induced Ca^2+^_i_ mobilisation was not dependent on signalling by Gα_s_, adenylyl cyclase-protein kinase A, Gα_i_/o-Gβγ, or protein kinase C, but did require PLC; and GHSR1a constitutive activity was not required as GHSR1a-F279L and -A204E did not prevent DRD1 non-canonical signalling (Kern *et al.* 2015). GHSR1a fusion proteins containing inducible homodimerisation domains showed that enhanced GHSR1a homodimerisation (and thus reduced GHSR1a availability to form GHSR1a-DRD1 heterodimers) correlated with reduced dopamine-induced Ca^2+^_i_ mobilisation. Moreover, BRET assays measuring Gα_q_-Gβγ in HEK293 cells overexpressing GHSR1a and DRD1 showed that SKF81297 attenuated signalling, consistent with DRD1 pre-coupling to Gα_q_ and agonist-induced dissociation of the heterotrimeric G protein complex; while pre-treatment with JMV2959 dose-dependently inhibited the SKF81297-induced decrease in BRET, indicating that GHSR1a is involved in the DRD1-Gα_q_ coupling. Dopamine-induced Ca^2+^_i_ activation was retained, but reduced, when DRD1 was expressed with GHSR1a mutants unable to couple to Gα_q_ (due to mutations in the third intracellular loop), confirming direct coupling of DRD1 to Gα_q_ (Kern *et al.* 2015).

A series of studies showed that GHSR1a is fundamentally important for DRD1-induced initiation of hippocampal synaptic plasticity and formation of hippocampal memory. Initially, phosphorylation of Ca^2^^+^/calmodulin-dependent protein kinase II and its target, the GluR1 subunit of α-amino-3-hydroxy-5-methyl-4-isoxazolepropionic acid receptor, which are involved in activation of synaptic plasticity, were shown to be activated at synapses by DRD1 activation. Phosphorylation of these proteins was lost in *Ghsr^−/−^* hippocampal slices. Next, exocytosis of glutamate receptors in hippocampal neurons was shown to be dependent on GHSR1a-DRD1 signalling. The transcription of genes involved in initiation of neuronal plasticity was also dependent on GHSR1a in hippocampal slices. Moreover, behavioural studies in mice showed: (i) DRD1-induced interference with prepulse inhibition, a measure of how well an animal can integrate and inhibit sensory information, is dependent on GHSR1a; (ii) Mice treated with SKF81297 exhibited a significant decrease in freezing behaviour in contextual and cued-fear conditioning apparatus, which was impaired when co-administered with JMV2959, consistent with allosteric interactions between GHSR1a and DRD1 in memory; and (iii) mice infused with SKF81297 continued to make correct choices in a T-maze alternation test, which was blocked by co-administration with JMV2959, consistent with enhanced memory when GHSR1a-DRD1 heteromers are activated (Kern *et al.* 2015).

Recently, a reduction in GHSR1a-DRD1 complexes in favour of establishment of GHSR1a-Aβ complexes was shown to correlate with Alzheimer’s disease (Tian *et al.* 2019). GHSR1a is overexpressed in hippocampal tissue from Alzheimer’s disease patients, and in the brains of 5xFAD mice, a model for the disease. This is particularly apparent in aged mice with heavy brain amyloidopathy with severe hippocampal lesions, in which GHSR1a forms complexes with amyloid beta (Aβ, the plaque deposits that are a hallmark of Alzheimer’s disease pathology), shown by proximity ligation assay (PLA). GHSR1a activity assays using fluorescein arsenical hairpin binder-based FRET in HEK293T cells showed Aβ has an antagonist-like effect on MK0677-mediated GHSR1a responses, and reduced GHSR1a-DRD1 complex formation (Tian *et al.* 2019). In GHSR1a-null, 5xFAD and double mutant GHSR1a-null/5xFAD mice there were similar reductions in synapse density, impaired stimulus-evoked LTP in hippocampal slices, and impaired spatial navigation in the Morris water maze test, indicating that the same pathways are affected in mice. Restoration of GHSR1a-DRD1 complexes, by simultaneous stimulation of GHSR1a and DRD1 with MK0677 and SKF81297 increased synaptic density in Aβ42-treated mouse hippocampal neurons; improved stimulus-evoked LTP, and restored miniature excitatory postsynaptic current frequency in 5xFAD mice; reversed memory defects in the Morris water maze test and reduced hippocampal synapse loss in 5xFAD mice (Damian *et al.* 2018). Thus, these studies demonstrate that disruption of GHSR1a-DRD1 complexes contributes to the synaptic abnormalities and behavioural impairments in mouse models of Alzheimer’s disease. The ability to rescue these complexes in hippocampal tissue, with consequent prolonged functionality, indicates that co-stimulation of GHSR1a-DRD1 could be an important preventative treatment for Alzheimer’s disease.

## GHSR1a interaction with dopamine receptor D2 (DRD2)

DRD2 is coupled to G_i__/o_ signalling pathways and hypothalamic dopamine signalling is important for basal regulation of food intake by influencing feeding frequency and volume (Meguid *et al.* 2000). DRD2 is reduced in the striatum of obese rats (which correlates with addictive behaviours), and knockdown of the receptor in rat striatum accelerates the onset of compulsive-like food seeking in rats with access to palatable high-fat food (Johnson & Kenny 2010).

Interactions between DRD2 and GHSR1a were first investigated in SH-SY5Y neuroblastoma cells stably overexpressing GHSR1a (SH-GHSR1). In contrast to native cells, in which activation of DRD2 with the selective agonist quinpirole induced G_i_ signalling and was not linked to calcium; SH-GHSR1 cells produced a dose-dependent increase in calcium, which was attenuated by the DRD2 antagonist raclopride and GHSR1a inverse agonist substance P. Quinpirole and ghrelin stimulation of calcium was also observed in primary hypothalamic neurons and HEK293 cells co-expressing the two receptors (Kern *et al.* 2012). Further studies in HEK293 cells with a range of inhibitors showed that these responses were mediated by G_i__/o_-PLC-IP3-Ca^2+^_i_ pathways, which likely involve direct association between Gβγ and GPCR kinase-2 (GRK2) (Kern *et al.* 2012). Co-expression of DRD2 with GHSR1a mutants that have low basal activity (F279L) showed dopamine could induce partial signalling, indicating that basal activity is unlikely to play a role, and that allosteric interactions between GHSR1a and DRD2 are likely more important (Kern *et al.* 2012). Heteromerisation was shown by TR-FRET in HEK293 cells and hypothalamic tissue, and cross-desensitisation, in which pre-treatment of cells with increasing concentrations of GHSR1a agonists MK0677 and ghrelin, or with DRD2 agonists dopamine or quinpirole, reduced dopamine-induced or ghrelin-induced calcium mobilisation, respectively. Additional evidence of DRD2-GHSR1a interactions were shown in animal models in which the DRD2 selective agonist cabergoline suppressed food intake in WT mice, whereas food intake in *Ghsr1^−/−^* mice was unaffected. Pre-treatment with a GHSR1a antagonist JMV2959 prevented the cabergoline effect on food intake (Kern *et al.* 2012).

A subsequent study investigated how GHSR1a and DRD2 oligomerise in styrene maleic acid copolymer membrane discs (SMALPs). SMALPs allow detergent-free solubilisation of membrane proteins within their native bilayer environment, which stabilises the extracted proteins (Dorr *et al.* 2016). Two- and three-colour FRET was used to show that heteromers contain two GHSR1a and two DRD2 protomers. Both receptors are associated with their respective G protein in these complexes, and G_i_ activation was more efficient in GHSR1a-DRD2 heteromers, and impaired in the absence of G_q_ (Damian *et al.* 2018). Further investigation using intramolecular time-resolved luminescence resonance energy transfer in HEK293 cells showed that GHSR1a-DRD2 association may affect the active conformation of Gα_i_. The authors propose a model in which GHSR1a exerts an allosteric effect on the adjacent receptors that modifies DRD2 conformation and its ability to trigger G protein activation (Damian *et al.* 2018).

Recently, a role for GHSR1a-DRD2 heteromers has been shown in the regulation of voltage-gated calcium channel currents in pre-synaptic terminals (Cordisco Gonzalez *et al.* 2020). Activation of Ca_V_2.2 channels in pre-synaptic terminals contributes to neurotransmitter release, and both ghrelin-mediated activation of GHSR1a and quinpirole activation of DRD2 have been shown to reduce Ca_V_2.2 channel currents and impair neurotransmitter release (Cordisco Gonzalez *et al.* 2020). DRD2 reduces Ca_v_2.2 in part by direct inhibition by Gβγ (Kisilevsky & Zamponi 2008). Studies in cultured hypothalamic neurons showed that co-expression of GHSR1a and DRD2 reduced the quinpirole-induced DRD2-mediated inhibition of Ca_V_2.2 currents (Cordisco Gonzalez *et al.* 2020). Further studies in transfected HEK293T cells confirmed that GHSR1a co-expression with DRD2 reduced the dopamine-induced inhibition of Ca_V_2.2 currents, which was blocked when cells were pre-incubated with the GHSR1a inverse agonist substance P or with dominant-negative G_q_. However, co-expression with MAS-GRK2-ct, a peptide that sequesters Gβγ from the G protein complex, had no effect on GHSR1a-mediated reductions in DRD2-mediated Ca_V_2.2 inhibition (Cordisco Gonzalez *et al.* 2020). The authors concluded that the Gβγ role in dopamine-mediated inhibition of Ca_V_2.2 is lost when GHSR is co-expressed, likely due to preferential coupling of Gβγ to G_q_ rather than G_i_, and reduces the ability of Gβγ to bind to Ca_V_2.2 (Cordisco Gonzalez *et al.* 2020). A subsequent study showed that LEAP2 blocks the GHSR1a-D2R co-expression effects on Ca_V_2.2 in transfected HEK293T cells (Mustafa *et al.* 2021). Using FRET, the authors showed that LEAP2 does not disrupt the formation of the GHSR-D2R heteromer, but stabilises GHSR1a in an inactive conformation (Mustafa *et al.* 2021). These studies provide evidence that targeting GHSR1a and DRD2 heteromers could allow selective fine-tuning of DRD2 signalling as neurons expressing DRD2 alone will be unaffected, which could improve selectivity of drug treatments and reduce their side effects (Kern *et al.* 2012).

## GHSR1a interaction with GHS-R1b

GHSR1b is a naturally occurring, highly expressed, but inactive truncated splice variant of GHSR1a (Gnanapavan *et al.* 2002, Navarro *et al.* 2016). However, GHSR1b can interact with GHSR1a to exert a dominant-negative effect on its signalling (Leung *et al.* 2007). In 2007, GHSR1a was reported to form homodimers and heterodimers with GHSR1b using saturation BRET and immunoprecipitation in HEK293 cells overexpressing the two proteins (Leung *et al.* 2007). GHSR1b had low, or absent, affinity for ghrelin; while constitutive activity, measured by radioactive IP production, was reduced in GHSR1a-GHSR1b complexes, although ghrelin-induced IP production was still present (Leung *et al.* 2007). The ability to phosphorylate extracellular signal-regulated kinase (ERK1/2) was unaffected by GHSR1b. Using ELISA, the authors showed a reduction in total GHSR1a and cell surface expression, indicating that GHSR1b may retain GHSR1a intracellularly, with confocal microscopy showing that this may be in nuclear or peri-nuclear locations (Leung *et al.* 2007). A subsequent study by the same group using sucrose-density gradients and saturation BRET showed GHSR1a-GHSR1b complexes form in the endoplasmic reticulum (ER) rather than the nucleus, and ERK1/2 signalling from these fractions declined when GHSR1b was co-transfected (Chow *et al.* 2012). These studies suggest the possibility that GHSR1a can signal from other locations than the plasma membrane, although this latter observation requires confirmation in further studies. The authors conclude that GHSR1a homodimerisation is a prerequisite for receptor trafficking to the cell surface, and that in the presence of GHSR1b, GHSR1a must compete to form functional dimers. The binding of GHSR1a and GHSR1b leads to a reduction in the activation of PLC, yet upon maximal stimulation by its ligand, GHSR1a can express sufficiently at cell surfaces to mediate signalling (Chow *et al.* 2012).

Another research group also examined GHSR1a-GHSR1b heterodimerisation by reconstitution in lipid nanodiscs (Mary *et al.* 2013). These studies confirmed that ligand-binding to GHSR1b is absent, indicating the importance of the TM6 and TM7 domains for ligand recognition, which was subsequently confirmed by receptor structural analyses (Shiimura *et al.* 2020); while GHSR1a-GHSR1b heterodimers can bind to a ligand (Mary *et al.* 2013). In contrast to similar signalling properties between GHSR1a monomers and homodimers, dimerisation with GHSR1b in lipid discs: abolished β-arrestin recruitment; prevented basal Gq activation; and was unable to mediate ghrelin-induced signalling (Mary *et al.* 2013). Finally, the impact of dimerisation on the receptor structure was explored using bimane labelling of TM7 as a fluorescent reporter to explore changes in GHSR1a conformation. In the absence of ligand, bimane emission properties were similar whether GHSR1a was monomeric, homodimeric, or heterodimeric, implying that dimerisation may not primarily affect the basal conformational state of the ghrelin receptor (Mary *et al.* 2013). In addition to the G protein heterotrimer, the ligand-free GHSR1a monomer was associated with a significant change in bimane emission intensity consistent with stabilisation of the active conformation of the receptor that is responsible for GHSR1a’s high constitutive activity (Mary *et al.* 2013). The GHSR1a homodimer had intermediate emission spectra compared to the monomer, which has previously been shown for leukotriene-B4 receptors and was attributed to asymmetry in the dimer, with only one of the two protomers in the fully active state (Damian *et al.* 2006). The GHSR1a-GHSR1b heterodimer, in contrast, showed no change in emission, indicating that the truncated receptor prevents GHSR1a from undergoing Gq-induced conformational changes, and explains the loss of constitutive and agonist-induced activity observed for these heterodimers (Mary *et al.* 2013).

These studies identified two mechanisms by which GHSR1b may have a dominant-negative effect on GHSR1a: (i) intracellular retention and loss of plasma membrane expression and (ii) an allosteric mechanism that prevents conformational changes required for GHSR1a signalling. A subsequent study by a third research group examined the GHSR1a-GHSR1b heterodimer in further detail, this time also addressing the physiological effect of such heterodimerisation (Navarro *et al.* 2016). Using overexpressed receptors in HEK293T cells, co-localisation of GHSR1a and GHSR1b in intracellular structures was confirmed and GHSR1b expression reduced the amount of GHSR1a in the plasma membrane. However, GHSR1b did not reduce total GHSR1a expression, nor did it prevent GHSR1a-GHSR1b heterodimer expression in the plasma membrane (Navarro *et al.* 2016). Label-free dynamic mass redistribution assays were used to examine GHSR1a-GHSR1b signalling and showed ghrelin-induced cAMP signalling was reduced by inhibition of G_i__/o_. Moreover, these assays showed that low expression of GHSR1b can potentiate GHSR1a-mediated G_i__/o_ signalling. A similar phenomenon was seen for ghrelin-induced increases in cytosolic calcium, phosphorylation of ERK1/2, and recruitment of β-arrestin, which were mediated by G_q_ when GHSR1a was expressed alone and absent from GHSR1b-only cells, but low expression of GHSR1b with GHSR1a resulted in potentiation of G_i_ signalling (Navarro *et al.* 2016).

In contrast to findings in HEK293T cells, in striatal and hippocampal neurons, there was an increase in G_s_-mediated elevations in cAMP. A DRD1 antagonist blocked this ghrelin-induced cAMP accumulation in striatal but not hippocampal neurons, indicating the involvement of DRD1 in the striatal GHSR1a-G_s_ coupling (Navarro *et al.* 2016). Studies in HEK293T cells demonstrated that DRD1 co-expression promoted a switch in GHSR1a-G protein coupling to G_s_, but only upon co-expression of GHSR1b. BRET assays confirmed complexes can form between GHSR1a and DRD1, but only in the presence of GHSR1b (Navarro *et al.* 2016) (Fig. 1). Thus, these studies imply that signalling by ghrelin is determined by the stoichiometry of GHSR1a to GHSR1b in the plasma membrane, and that findings of intracellular retention are due to artificially high expression of GHSR1b in some studies. This mechanism of G protein switching by GHSR1a, when co-expressed with GHSR1b, will need to be considered when designing compounds targeting heterodimers. Moreover, these studies demonstrate the importance of replicating studies in physiologically relevant cells in which receptor stoichiometries are accurate and tissue-specific effects can be observed.

## GHSR1a interaction with neuronal sigma-1 receptor (σ1R)

The σ1R is a two transmembrane pass protein that is preferentially expressed at the ER and acts as a molecular chaperone. The native ligand for the receptor is unknown; however, cocaine can bind to the receptor (Lever *et al.* 2016). As cocaine suppresses appetite, and GPCRs including DRD1 and DRD2 have been shown to interact with σ1R, heteromerisation between GHSR1a and σ1R was investigated. Using HEK293T cells, GHSR1a and σ1R were shown to colocalise using immunocytochemistry and BRET assays, with GHSR1a TM1 and TM2 most likely to interact with σ1R, identified using BiFC and cell-penetrating peptides (Aguinaga *et al.* 2019). Computational modelling using molecular dynamics simulations indicated that a single GHSR1a protomer would be unable to interact with both σ1R at TM1/2 and the G protein at the intracellular receptor face, and therefore it is likely that dimers of GHSR1a (or GHSR1a-GHSR1b) are required (Aguinaga *et al.* 2019). Cell treatment with cocaine, or the σ1R agonist PRE-084, increased σ1R-GHSR1a co-localisation and suppressed GHSR1a-mediated cAMP reductions and phosphorylation of ERK1/2. Disruption of interactions between σ1-receptor and GHSR1a by TM1 interference peptide blocked the effect of cocaine on GHSR1a function. As cocaine’s main effects on the CNS are exerted in the striatum, studies were repeated in primary striatal neurons, which showed enhanced σ1R-GHSR1a interactions by PLA and impaired GHSR1a-mediated signalling on pre-treatment with cocaine or PRE-084 (Aguinaga *et al.* 2019).

While these studies indicate that σ1R-GHSR1a interactions may occur in the striatum and could contribute to the mechanism by which cocaine suppresses appetite, further investigations are required to establish whether cocaine affects ghrelin-mediated physiological functions *in vivo*. As σ1R and GHSR1a form interactions with both DRD1 and DRD2, it is possible that complex heteromers could form between different combinations of these proteins, and the concentration of ligand that relevant tissues are exposed to could determine the formation of these heteromers and their effects on appetite and reward pathways.

## GHSR1a interaction with oxytocin receptor

The oxytocin receptor (OTR) is a G_q_-coupled receptor that is expressed on reproductive tissues and brain regions including the hypothalamus, hippocampus, and amygdala (Gimpl & Fahrenholz 2001). The oxytocin neuropeptide acts as the primary ligand for the receptor and has well-described functions in bonding, reproduction, and stress responses (Gimpl & Fahrenholz 2001). Oxytocin has also been shown to regulate other hormones including ghrelin, although these studies disagree on whether oxytocin enhances or inhibits ghrelin secretion (Vila *et al.* 2009, Iwakura *et al.* 2011).

A single study has investigated interactions between GHSR1a and OTR (Wallace Fitzsimons *et al.* 2019). Co-localisation between OTR and GHSR1a was shown by confocal microscopy, and close proximity of the receptors was confirmed by FRET in HEK293 cells. Additionally, the receptors are expressed in the same structures in hypothalamic and hippocampal postnatal rat neuronal cultures, indicating that the receptors could theoretically interact in physiological systems (Wallace Fitzsimons *et al.* 2019). Oxytocin-induced Ca^2+^_i_ signalling was reduced in cells co-expressing both OTR and GHSR1a, while GHSR1a responses remained normal (Wallace Fitzsimons *et al.* 2019). This was prevented by pre-treatment of cells with the GHSR1a antagonist JMV2959. Confocal imaging showed that stimulation with ligands for either receptor-induced increased internalisation of OTR and GHSR1a, indicating that the receptors may co-internalise (Wallace Fitzsimons *et al.* 2019).

While this study provides some evidence that OTR and GHSR1a receptors may interact, a number of questions remain, which require further study to address. This includes determining how increased co-internalisation of both OTR and GHSR1a results in impaired signalling of only the OTR, as well as investigating the physiological relevance of such an interaction.

## GHSR1a interaction with prostanoid receptors

Prostanoid receptors are GPCRs that bind the arachidonic acid metabolites prostaglandins and thromboxane A_2_. Their functions include relaxation and contraction of smooth muscle, particularly during the inflammatory process and in platelet activation (Narumiya *et al.* 1999). Ghrelin and GHSR1a have also been described to regulate inflammatory responses and vasodilation. GHSR1 mRNA is present in vascular smooth muscle cells, cardiomyocytes, monocytes, T-lymphocytes, neutrophils, and macrophages; ghrelin increases blood flow and decreases arterial pressure in humans; and ghrelin inhibits the production of leptin-induced proinflammatory cytokines, while leptin upregulates GHSR1a expression on human T-lymphocytes (Dixit *et al.* 2004, Waseem *et al.* 2008). This suggests the existence of a reciprocal regulatory network by which ghrelin and leptin control immune cell activation and inflammation, coupling the metabolic axis to the immune system (Dixit *et al.* 2004).

These findings led to the hypothesis that GHSR1a and prostanoid receptors may cross-talk or interact to regulate each other’s activity. One study investigated the interaction between transiently transfected GHSR1a and the prostaglandin E_2_ receptor subtype EP_3-I_ and thromboxane prostanoid A2 receptor, TPα, in HEK293 cells (Chow *et al.* 2008). GHS-R1a/EP_3-I_ and GHS-R1a/TPα heteromers were demonstrated by saturation BRET, and evidence of co-immunoprecipitation between the receptors led the authors to state complexes are stable (Chow *et al.* 2008). Co-transfection of GHSR1a and prostanoid receptors reduced GHSR1a signalling via three mechanisms: decreased total GHSR1a expression, increased intracellular localisation of GHSR1a, and reduced GHSR1a constitutive activity. These findings require replication in more physiologically relevant cell lines or whole animal models; however, this mechanism could explain findings that ghrelin and prostacyclins have opposite actions in smooth muscle in the regulation of inflammation.

## Conclusions

GHSR1a has been shown to interact with proteins with diverse functions in multiple tissues, and understanding how this modifies signalling could provide novel strategies to target receptors in specific locations. However, many of these studies rely on overexpression of proteins in cell lines and reported heterodimers could be artefacts of receptor crowding and random collisions. Investigations in physiologically relevant tissues, assessment of endogenous proteins, and replication in independent laboratories are required for many of these reported interactions before advancement to drug discovery. GHSR1a interacting proteins do, however, represent a potentially exciting new target for appetite regulation, Alzheimer’s disease, insulin secretion, and inflammation.

## Declaration of interest

The authors declare that there is no conflict of interest that could be perceived as prejudicing the impartiality of this review.

## Funding

C M G is in receipt of research funding in the form of an Academy of Medical Sciences http://dx.doi.org/10.13039/501100000691 Springboard Award (Ref: SBF004|1034), which is supported by the British Heart Foundationhttp://dx.doi.org/10.13039/501100000274, Diabetes UK http://dx.doi.org/10.13039/501100000361, the Global Challenges Research Fund http://dx.doi.org/10.13039/100016270, the Government Department of Business, Energy and Industrial Strategy and the Wellcome Trust http://dx.doi.org/10.13039/100010269.
